# P-1538. Analyzing Trends and Outcomes of Clindamycin Resistant Invasive Group A Streptococci Infections

**DOI:** 10.1093/ofid/ofae631.1706

**Published:** 2025-01-29

**Authors:** Tamara Jordan, Megha Jagannathan, Daniel Kinsey, Rachel M Kenney, Michael Veve, Geehan Suleyman, Anita Shallal

**Affiliations:** Henry Ford Hospital, Detroit, Michigan; Henry Ford Hospital, Detroit, Michigan; Henry Ford Hospital, Detroit, Michigan; Henry Ford Hospital, Detroit, Michigan; Henry Ford Health, Detroit, Michigan; Henry Ford Health, Detroit, Michigan; Henry Ford Health, Detroit, Michigan

## Abstract

**Background:**

Streptococcus pyogenes (Group A Streptococci; GAS) is a gram-positive bacterium that is a leading cause of life-threatening infections. For invasive infections, IDSA recommends high-dose penicillin and clindamycin (DA). However, increasing resistance to DA has been reported. The aim of this study was to determine the prevalence of DA-resistant GAS and evaluate if DA resistance was associated with worse outcomes.

Table

**Figure:**
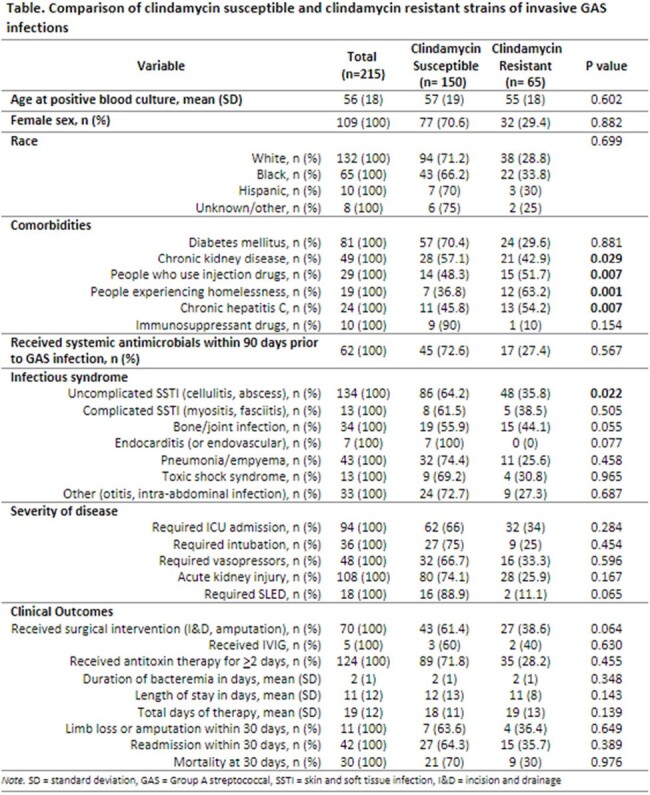

Comparison of clindamycin susceptible and clindamycin resistant strains of invasive GAS infections

**Methods:**

This was a retrospective cohort study from June 2013 to December 2023 across a five-hospital health system in Southeast Michigan of patients with positive blood cultures for GAS who received DA for anti-toxin therapy identified through Microsoft SQL. Children, polymicrobial bacteremia, incomplete data, receiving linezolid empirically, or those who died within 48 hours of admission were excluded. Patients with DA susceptible (DA-S) GAS isolates were compared to patients with DA resistant (DA-R) GAS isolates. Variables included demographics, infection characteristics, microbiologic data, therapy, and clinical outcomes.

Figure

**Figure:**
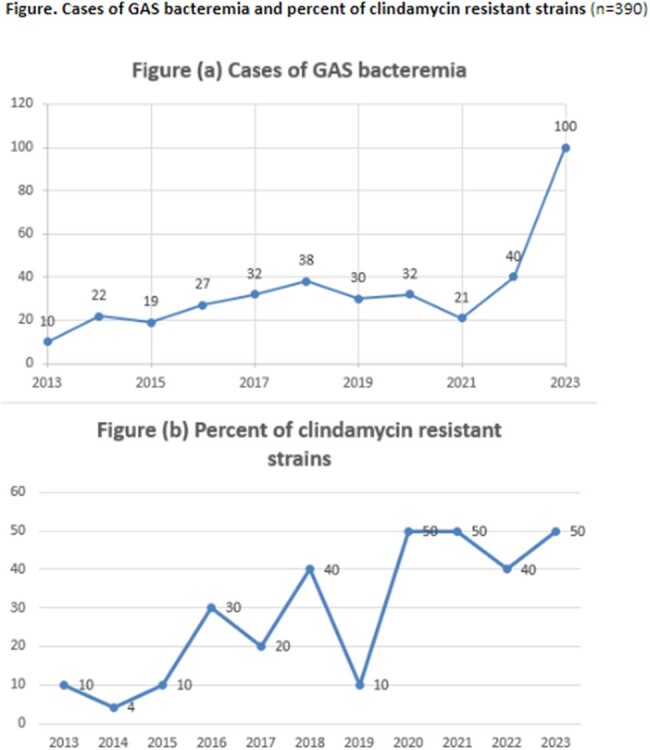

Cases of GAS bacteremia and percent of clindamycin resistant strains

**Results:**

390 cases were reviewed, and 215 were included in the cohort study [Table]. There was no difference in age, sex, or race among the groups. People who use injection drugs (51.7% vs 48.3%, p=0.07), people experiencing homelessness (PEH; 63.2% vs 36.8%, p=0.001), and chronic hepatitis C infection (HCV; 54.2% vs 45.8%, p=0.007) were more prevalent in the DA-R group; chronic kidney disease was more frequent in the DA-S group (57.1% vs 42.9%, p=0.029). Uncomplicated SSTI was more common in the DA-S group (64.2% vs 35.8%, p=0.022). There was no significant difference in the severity of illness, duration of bacteremia, surgical management, treatment duration, length of stay, readmission or mortality between the two groups. There was an increased incidence of invasive GAS infections beginning in 2022, and 50% of isolates were DA-R in 2023 [Figure].

**Conclusion:**

Although there is an increase in DA-R in invasive GAS infections, there was no significant difference in outcomes among patients with DA-R and DA-S who received standard of care treatment in addition to DA antitoxin therapy. Further research is needed to determine the clinical significance of these findings to inform optimal therapy for these groups.

**Disclosures:**

**Rachel M. Kenney, PharmD, BCIDP**, Medtronic Inc: Spouse is an employee, stockholder

